# Confidence in Health-Services Availability during Disasters and Emergency Situations—Does it Matter?—Lessons Learned from an Israeli Population Survey

**DOI:** 10.3390/ijerph16193519

**Published:** 2019-09-20

**Authors:** Odeya Cohen, Stav Shapira, Limor Aharonson-Daniel, Judith Shamian

**Affiliations:** 1 Nursing Department, Recanati School for Community Health Professions, Faculty of Health Sciences, Ben-Gurion University of the Negev, P.O. Box 653, Beer-Sheva 84105, Israel; 2 School of Public Health, Faculty of Health Sciences, Ben-Gurion University of the Negev, P.O. Box 653, Beer-Sheva 84105, Israel; stavshap@bgu.ac.il (S.S.); limorad@bgu.ac.il (L.A.-D.); 3 PREPARED Center for Emergency Response Research, Ben-Gurion University of the Negev, P.O. Box 653, Beer-Sheva 84105, Israel; 4 International Council of Nurses ICN President Emerita, FAAN, 88 Rockford Rd, Toronto, ON M2R3A7, Canada; shamianjudith@gmail.com

**Keywords:** CCRAM, community resilience, emergency management, health services availability, public health in disasters

## Abstract

The association between health and community resilience is well established in the literature. However, maintaining continuity of healthcare services during emergencies, and their contribution in the context of community resiliency have not been sufficiently studied. This study aims to explore the relationship between the public’s confidence in the availability of healthcare services during and following emergencies, and community resilience. A cross-sectional study was conducted among 3478 Israeli adults, using the Conjoint Community Resilience Assessment Measurement (CCRAM) tool. Associations between confidence in health services availability during emergencies, socio-demographic variables, and community resilience as measured by the CCRAM score were analyzed. The results revealed that confidence in the availability of health services positively correlated with community resilience score (r(3377) = 0.580, *p* < 0.001), and that it contributed significantly to increasing resilience (OR = 2.67, 95% CI (2.4–2.9), *p* < 0.001). Maintaining continuity of healthcare services during emergencies has effects beyond the provision of medical treatment. For instance, the confidence of the population in the availability of these services contributes to community resilience. In turn, this finding can be translated into practical resilience building actions and to facilitate community health.

## 1. Introduction

Community resilience is a multifaceted and cross-disciplinary concept often used in the context of emergencies to describe the ability to cope, mitigate, and rebound quickly after the event [1,2]. The association between community resilience and public health has been widely discussed in the literature in relation to both routine and emergency situations [2,3,4,5]. During routine times, community resilience is interpreted in the light of the definition of public health as a state of complete physical, mental, and social wellbeing (and not merely the absence of disease) [6]. However, in the context of public health emergency management, community resilience takes on a broader significance depending on the different stages of the event. Prior to the occurrence of an emergency situation, community resilience facilitates the emergence of an organizing framework for managing the preparedness efforts of public health institutions [7]. 

In order to fully comprehend the meaning of community resilience, we must first clearly define the term “community”. According to MacQueen et al., community is “a group of people with diverse characteristics who are linked by social ties, share common perspectives, and engage in joint action in geographical locations or settings” [8]. The subjective perception of a community is usually explored through attitudes of bonding (trust, connection, and engagement) with other community members, as well as with local institutions and systems (e.g., local leadership, local education system, local healthcare providers) [1]; for the most part, these are considered as protective factors in crisis situations [3]. 

Enhancement of certain community resilience attributes (e.g., social connectedness, public-private partnerships) has been shown to contribute to health system preparedness for emergencies [9]. Other community resilience elements, such as effective risk communication and connected leadership, likewise contribute significantly to an effective response [10] and have been shown to be substantial components of public health actions and relief efforts during crisis [11]. The incidence of different health conditions (such as Post-Traumatic Stress Disorder (PTSD)) among individuals who have experienced emergencies is affected by their community’s social and structural capital, which is also a component of community resilience [12]. This has implications for public health providers engaged in supporting the affected population following a disaster or an emergency and emphasizes the salience of community resilience in the complex dynamic of the emergency recovery process [13]. A recent publication which examined synergizing public health concepts with the Sendai framework for disaster risk reduction [14], concluded that directing actions both at the individual and at the community level to strengthen disaster resilience may be more effective than traditional disaster risk reduction strategies that focus solely on the behavior of individuals, particularly in addressing health inequities [15]. 

In 2011, the U.S. Centers for Disease Control and Prevention (CDC) published a set of public health preparedness capabilities designed to serve as the basis for state and local strategic planning. Community resilience was included as one of these capabilities. Major issues identified in relation to the process of building community resilience were the need for pre-assessment of potential loss and disruption of essential public health services (e.g., provision of healthcare), and planning for continuity of operations for these services during response and recovery situations [5]. The importance of the latter was demonstrated when Hurricane Katrina struck the Gulf Coast of the U.S., destroying many hospitals and clinics located in its path and, along with them, the hope of continuity of care for many of the storm’s victims. The implications for the physical and emotional stress suffered by these individuals and for their recovery were profound and far-reaching [16]. Apart from providing necessary medical treatment for casualties, healthcare institutions, including hospitals and community clinics, are often perceived as a source of social support by disaster-affected communities, whom they serve as providers of information and guidance and suppliers of basic needs such as water, food, and shelter [17,18]. Healthcare personnel are known to be highly committed to care for the injured or ill even in situations that may endanger them or their families—therefore, they merit high levels of public trust which also serves as the foundation for their confidence in availability of healthcare [19]. It is hardly surprising, therefore, that some researchers from the field of public health emergency management recommend that these institutions be placed at the core of community resilience [20]. Based on these findings, it can be assumed that the confidence that local healthcare services will continue to operate during an emergency will have a positive impact on the level of community resilience in the population. 

Reliable data about the levels of community resilience in the population and the factors that may affect community resilience during the pre-emergency period could be of vital importance for authorities and decision makers, among them public health agencies. Such data would facilitate the development of evidence-based intervention programs for strengthening the community and addressing specific health needs or inequities [2,15]. The conjoint community resiliency assessment measure (CCRAM) is a population-based measure, recognized as a valid tool for assessing community resilience by household sampling [21,22]. CCRAM encompasses the various components of a community’s resilience, identified through a statistical process and also anchored in the professional literature surrounding the concept of community resilience. This study is part of a set of studies aiming for elucidating the factors associated with community resilience and the characteristics of community resilience in different sub-populations and aspects [1,23]. 

The aim of this study is to explore the relationship between the public’s confidence in the availability of healthcare services during an emergency, and community resilience levels among residents of small (up to 10,000 people) and midsize communities (up to 50,000). It is hypothesized that a positive relationship exists between the public’s confidence that local healthcare services will continue to function during an emergency and the average community resilience score.

## 2. Materials and Methods

A cross-sectional survey using the CCRAM tool to measure community resilience was carried out during a baseline period with no major emergency responses. CCRAM is a self-report questionnaire with 28 items, the first 21 of which comprise the community resilience score (see Table A1 for items description). Responses are rated on a 5-point Likert scale. Seven items (22–28) provide additional important information on issues related to community resilience, one being confidence in the availability of health services during emergencies. The respondent is requested to rank his/her agreement with the statement: “The health services in my town will continue to function appropriately in an emergency situation”. 

### 2.1. Data Collection

Data were collected between 2012 and 2014. A multi-level stratified sample was obtained by partitioning into small communities (up to 10,000 residents) and midsized towns (up to 50,000 residents), based on definition of the Israeli Central Bureau of Statistics [24]. Eligibility criteria for study inclusion encompassed participants over the age of 18, understanding the questionnaire (the study was conducted with cultural sensitivity, the questionnaire was available in four languages). Door-to-door surveys were the means of data collection. Surveyors visited randomly selected addresses, in addition to which, electronic questionnaires were sent out in small communities with a self-contained electronic mailing list, using Qualtrics (www.qualtrics.com), web-based survey software. Prior to beginning, permission was sought and obtained from the Institutional Review Board (IRB), Faculty of Health Sciences at Ben-Gurion University of the Negev. The questions were preceded by an introduction that listed study objectives, stating the participation was voluntary and participants could withdraw at any point, and that anonymity was guaranteed. In accordance with IRB guideline, filling in the questionnaire signified informed consent. 

### 2.2. Statistical Analysis

Cronbach’s alpha was used to examine CCRAM reliability, and Pearson correlation coefficients to examine the association between CCRAM factors and confidence in health service availability during emergencies, and between the latter and several background variables. An independent *t*-test and ANOVA, followed by *post hoc* tests, served to examine differences in mean CCRAM scores and confidence in health services availability among sub-groups of participants. For some analyses, CCRAM score was classified into two levels based on the population median community resilience score of 3.524: low, 1–3.523 (*n* = 1730), vs. high, ≥3.524 (*n* = 1663). In order to explore the factors associated with levels of CCRAM score, hierarchical logistic regression (HLR) was performed. The HLR modeled the dependent variable, namely the categorical CCRAM score. The model was run with two blocks: (1) socio-demographic variables—gender, age, being in a permanent relationship, education, physical or mental disability, community type, reported income level, and volunteering with the community emergency response team (CERT); (2) confidence in the availability of health services during emergency situations. In this analysis, confidence in health-service availability was added to the model after the socio-demographic variables in order to enable examination of the net contribution beyond the socio-demographic characteristics. Odds ratios are used in HLR to estimate the strength of association or non-independence between a binary and a categorical variable [25]. In this study we used odds ratios to describe the effect of certain variables on the CCRAM score: the greater the odds ratio, the higher the score. The estimate covariance matrix was reviewed to verify reasonable tolerance. 

In addition, classification probabilities for low/high CCRAM scores from the two steps of the HLR were saved. These were then submitted to receiver operating characteristic (ROC) analysis, which describes the accuracy and diagnostic value of the CCRAM tool in terms of its sensitivity-specificity trade-off [26]. The ROC curves of each step, together with the area under the curve (AUC) in the HLR also served to support a contribution of confidence in health services to the CCRAM score and the quality of the model fit. IBM SPSS^®^ version 24.0 (IBM Corp, Armonk, NY, USA), the statistical package for the social sciences, was used for data analysis.

## 3. Results

**Figure 1:**
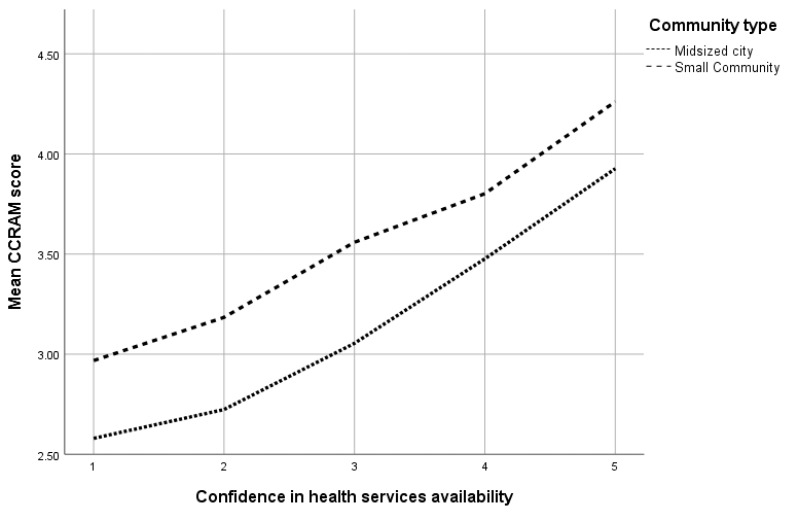
Associations between mean CCRAM scores and confidence in health services availability in accordance with community type.

The participants in the study were 3478 adults (mean age 40.7 years, range 18–93, SD 14.42 years) living in small (*n* = 1813, 52.1%) and midsize communities (*n* = 1645, 46.4%). About 60% of the responders defined themselves as secular (*n* = 2019), and 87% stated that they had no physical or mental disability (*n* = 3024). No significant difference was found in confidence in health services availability between healthy respondents and those who defined themselves as having a physical or mental disability. Table 1 presents the major study population characteristics. Figure 1 presents associations between CCRAM scores and confidence in health services availability in accordance with the community type. The CCRAM questionnaire showed high reliability (α = 0.933). Detailed information is presented in Table A1 and Table A2.

Confidence in the availability of health services during emergency situations and the CCRAM score were positively correlated (r(3377) = 0.580, *p* < 0.001), and positively associated with each CCRAM factor. The relationship with leadership was (r(3376) = 0.619, *p* < 0.001), collective efficacy (r(3375) = 0.452, *p* < 0.001), preparedness (r(3374) = 0.496, *p* < 0.001), place attachment (r(3375) = 0.320, *p* < 0.001), and with social trust (r(3366) = 0.311, *p* < 0.001). Table A3 displays associations between confidence in health services availability and individual CCRAM items. 

### Hierarchical Logistic Regression (HLR)

Hierarchical logistic regression was conducted to explore the contribution of confidence in health services availability during emergencies to community resilience level. Table 2 presents the results of the last step of the HLR. Significant socio-demographic variables were identified by this model. Living in a small community had an odds ratio of 4.054 (95% CI 3.39–4.85) as compared with the reference group of midsized towns. Low income level as compared with average income had an OR of 0.701 (95% CI 0.55–0.89). The contribution of confidence in health services availability independently of the socio-demographic variables was statistically significant, with a change in the −2 log likelihood of 520 with *p* < 0.001. The regression coefficients are displayed in Table A2.

**Figure 2:**
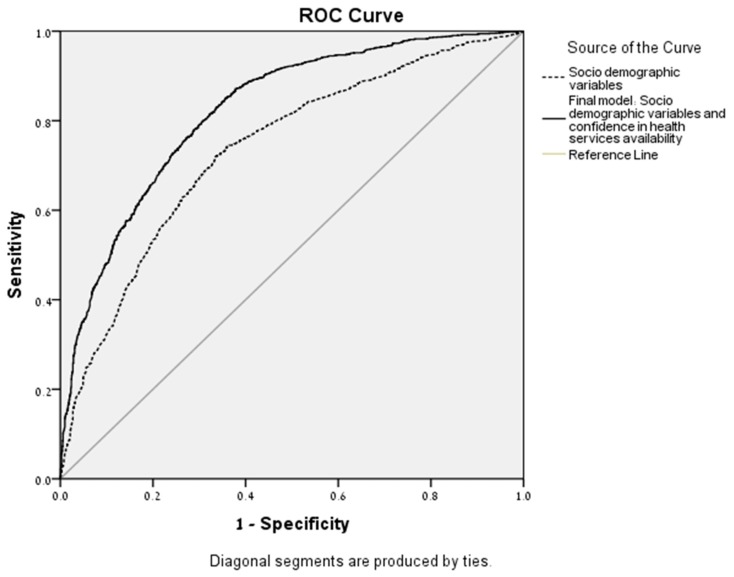
Receiver operating characteristic (ROC) curves of the socio-demographic variables compared with final model including confidence in health services availability during emergencies.

Classification probabilities of low/high perceived community resilience levels resulting from the HLR model were further analyzed using ROC analysis to assess the diagnostic value of CCRAM. Two curves were plotted, (1) socio-demographic variables and (2) socio-demographics and confidence in health services availability (Figure 2). The two models demonstrated significance beyond the chance-alone reference curve. Area under the curve (AUC) for socio-demographic variables was 0.737 (SE = 0.009, *p* < 0.001; 95% CI (0.719–0.754)), while for socio-demographic variables and confidence in the availability of health services—AUC = 0.824 (SE = 0.08, *p* < 0.001; 95% CI (0.810–0.839)). The contribution of trust in health services availability independently of socio-demographic variables increased the AUC by 0.09, and there was no overlap of the 95% CI, indicating significant contribution of confidence in health services availability to the CCRAM score.

## 4. Discussion

The importance of community resilience in the public health domain, has been emphasized repeatedly since 2009 when it was defined by U.S. Federal, state, and local levels in the National Health Security Strategy [27]. In 2015, the European Union introduced the Resilience Marker in all the humanitarian projects it funds. This marker defines ways to reduce disaster risks and to strengthen people’s coping capacities including the need for strengthening local healthcare systems [28].

The current study explores the relationship between residents’ trust in the availability of healthcare services during an emergency, and their community resilience score in a population that is prone to emergency situations. The fact that no significant difference was found in confidence of health services availability between healthy respondents and those who were not (See Table A1), can indicate the perceived importance of health care services in both healthy and unhealthy people.

The study replicated previous findings on characteristics of community resilience [29,30]: no differences were noted in the average CCRAM score by gender. Higher resilience was identified in people in a permanent relationship, in those residing in smaller communities (which are more cohesive), and in CERT volunteers. Lower community resilience was found in people with income below average and in those with disabilities. These results were later strengthened by a Hierarchical logistic regression model of a categorized CCRAM score. The model showed that living in a small community had the highest odds ratio of 4.054 (95% CI 3.39–4.85) as compared with the reference group of midsize towns. Although not all communities function similarly, our results, as presented in Figure 1, indicate that there are equal trends in associations between community resilience scores and confidence in health services availability among different types of communities. 

While the above-mentioned results were reaffirmations of previous studies [29,30], the original and significant findings of the current study are that the participants’ trust in the availability of health services during emergency situations was positively correlated to their community resilience level. This result means that where the public is informed and confident that there will be access to health services after a disaster, the community is more resilient. Furthermore, when examining the various CCRAM items [23], strongest significant relationships were found between trust and faith of leadership functioning (see Table A3). This can usefully be translated into real-world, practical recommendations regarding actions that would enhance resilience and public health preparedness capabilities. 

Strong local public healthcare systems are the cornerstone of an effective public health response [5]. Public health preparedness capabilities have therefore been defined as the basis for local strategic planning. One of the capabilities mentioned in strategic plans is community resilience. The process of building community resilience includes planning for continuity of essential public health services during emergency response and recovery situations [5]. 

In this context, Morton and Lurie stress the important role of social capital in a community’s recovery and mention the LA model which includes expanding upon the community engagement skills of emergency responders, increasing the preparedness knowledge of public health workers and using mapping tools to identify potential areas of vulnerability in community resilience [31]. Among the many factors mentioned, the continued availability of healthcare services was not identified by their research. We would thus like to stress this important aspect and suggest adding it to all strategic plans as a practical approach to achieving functional public health emergency management programs. 

At the outset of this study, we hypothesized that a relationship would be found between the public’s confidence that local healthcare services will continue to function during an emergency and their average community resilience score. We did not anticipate the scope of this relationship. Evidence shown above demonstrated the significance of the healthcare system to community resilience, and at the same time, it identifies trust in leadership (regarding functional continuity in emergency situations and infrastructure) which has been referred to as a crucial element in the past [32]. Supply of healthcare during an emergency situation may have more implications than provision of healthcare to civilians in routine times. This significance is reflected in the strong correlation found between the confidence that healthcare facilities will be available and the factors of community resilience, an idea that echoes that of the hospital as a safe haven for the population [17]. The capacity to provide medical treatment and health support has a positive indirect influence on a community’s ability to cope with stressful conditions.

While the literature has identified factors that are likely to be correlated with achieving resilience for communities, these domains have been rather broad and lack the specificity required for implementation. The relationship between health and resilience has been explored in the past, and good health prior to disasters has been reported to support greater resilience in the disaster setting [33,34]. In the exploration of community resilience, we aimed at finding the core factor, the factor that when enhanced, will have a large effect on the augmentation of resilience [29]. From the findings above, it seems that the availability of healthcare services during an emergency situation may be a factor that can be translated into practical, evidence-based, resilience building actions. Resilience is a concept that constitutes the point of interface between routine and emergency. Resilience-building actions are often conducted during routine. Our findings suggest a possible outline for enhancing community resilience through two directions: (1) strengthening the relationship between local healthcare services and local population through strategies that consider the unique characteristics and needs of different sub-groups (e.g., young children, people with disabilities, chronically ill patients etc.); (2) development of response plans directed at increasing the capacity of local healthcare services to maintain continuity of care during calamities. Such increased capacity is especially important in less-developed regions and especially when healthcare services may be deliberately targeted (e.g., in conflict zones) or severely harmed (e.g., during natural disasters). In these cases, response plans should include establishing a support network to provide tools for local healthcare professionals and enable them to operate independently and effectively despite the poor infrastructure. 

### Limitation and Further Studies

This cross-sectional study enabled us to identify associations but not causality. The effect of health services during emergencies on building community resilience could be explored in future longitudinal studies that will measure community resilience scores with actual availability of health services. This study was conducted during 2012–2014, and perceptions may have changed since then. Because electronic mailing lists were used to approach some of the study population, our ability to assess response rate was compromised, and this is another limitation of this study—there was no mechanism for ascertaining that an email arrived and if so, whether it was opened. Many more women than men responded, and their percentage was higher than what it is in the general population, a rate similar to that found in other studies; by and large, it seems that women, more than men, tend to respond to surveys [35]. As this study is a first step towards determining the correlation between confidence in the availability of health services during emergencies and community resilience scores; we recommend that further studies will focus on both policy as well as practical implications arising from this association.

## 5. Conclusions 

Confidence in continuity of health services during a state of emergency was found to be positively correlated with community resilience and could, therefore, be considered as a possible path for its enhancement. The implications for health policy are that maintaining healthcare services at the local level at a time of emergency may enhance the community resilience to disasters, facilitate a better recuperation and influence in many levels on the community health beyond the provision of medical treatment. Special attention should be given to communities where healthcare facilities were—or could be—directly targeted or severely harmed due to belligerence or a natural disaster. For such communities, response plans should include setting up a supporting network that provides tools for local healthcare professionals, enabling them to operate independently when outside assistance is curtailed. 

## Figures and Tables

**Table 1:** Major study population characteristics, mean conjoint community resiliency assessment measure (CCRAM) scores and confidence in health services availability during emergency situations.

Variable	*n*	%	CCRAM Score	*p*-Value (*t*-Test or ANOVA)	Confidence in Health Services Availability	*p*-Value (*t*-Test or ANOVA)
**Total**	3478	100	3.46		3.37	
**Gender**						
Female	2014	57.9	3.46	0.069	3.40	0.978
Male	1424	40.9	3.43		3.32	
**Family status**						
In a permanent relationship	2668	76.7	3.49	0.001	3.40	0.001>
Not in a permanent relationship	765	22.0	3.28		3.27	
**Community type**						
Midsize town (up to 50,000)	1615	46.4	3.14	<0.001	3.10	0.001>
Small community (up to 10,000)	1813	52.1	3.74		3.62	
**Income**						
Below average	804	23.1	3.26	<0.001	3.16	0.001>
About average	1195	34.4	3.47		3.36	
Above average	1354	38.9	3.52		3.50	
**CERT volunteer**						
No	2951	84.8	3.81	<0.001	3.69	0.06
Yes	474	13.6	3.38		3.32	
**Physical or mental disability**						
No	3024	86.9	3.46	0.001	3.38	0.124
Yes	424	12.2	3.33		3.29	
**Previous involvement in an emergency situation**						
No	1547	44.5	3.44	0.418	3.36	0.766
Yes	1257	36.1	3.47		3.35	

CERT—community emergency response team.

**Table 2:** Association between covariates and CCRAM score, logistic regression model.

	Odds Ratio	*p*	95% Confidence Interval	
Variable	(OR)		Lower	Upper
**Gender**				
Female	1			
Male	0.872	0.131	0.730	1.042
**Age (per year)**	1.001	0.878	0.994	1.007
**Permanent relationship**				
Yes	1			
No	0.772	0.023	0.617	0.964
**Physical or mental disability**				
No	1			
Yes	0.916	0.534	0.695	1.208
**Community type**				
Midsized city	1			
Small community	4.009	<0.001	3.358	4.786
**Income**				
Average	1			
Below	0.709	0.005	0.559	0.900
Above	0.823	0.059	0.673	1.007
**CERT volunteer**				
No	1			
Yes	2.120	<0.001	1.614	2.785
**Confidence in health services availability**	2.677	<0.001	2.433	2.946

CERT—community emergency response team; −2 log likelihood at step 1= 3714.68 (df = 8) and at step 2 = 3093.63 (df = 9); −2 log likelihood change of 2nd block with df = 1 is −621.05, Chi square *p* < 0.001.

## Appendix A

**Table A1:** Distribution of scores for individual CCRAM items.

No.	Phrase	Min	Max	Mean	SD
1.	The municipal authority functions well.	1	5	3.49	1.087
2.	There is mutual assistance and people care for one another.	1	5	3.64	1.111
3.	My community is prepared for an emergency situation.	1	5	3.26	1.070
4.	I am proud to tell others where I live.	1	5	4.16	0.997
5.	Good relationships exist between the various groups.	1	5	3.48	0.961
6.	I trust the local decision makers	1	5	3.18	1.126
7.	I can count on people in my community to help me in a crisis situation.	1	5	3.73	1.142
8.	Residents are aware of their roles in an emergency situation.	1	5	2.91	1.159
9.	I have a sense of belonging to my community.	1	5	3.94	1.057
10.	Residents in my community trust each other.	1	5	3.46	0.963
11.	In my community, appropriate attention is given to the needs of children.	1	5	3.58	1.062
12.	In my community, there are people who can help me cope with an emergency situation.	1	5	3.76	1.023
13.	There are sufficient facilities for public protection (e.g., shelters, etc.) in my community	1	5	3.07	1.212
14.	I remain in my community for ideological reasons.	1	5	2.97	1.416
15.	I have faith in my mayor’s ability to lead the transfer from routine to emergency management.	1	5	3.32	1.141
16.	I have faith in my community’s ability to overcome an emergency situation.	1	5	3.79	0.987
17.	My family and I are acquainted with the emergency system in my town (to be activated in times of emergency).	1	5	2.92	1.249
18.	I would be sorry to leave the town where I reside.	1	5	3.95	1.228
19.	The municipal authorities provide services fairly.	1	5	3.31	1.096
20.	The residents are greatly involved in the community’s activities.	1	5	3.33	1.041
21.	The residents of my community will continue to receive municipal services even in an emergency situation.	1	5	3.43	0.995
22.	I feel safe in my place of residence.	1	5	3.72	1.011
23.	The health services in my town will continue to function appropriately in an emergency situation.	1	5	3.39	1.096
24.	The information I receive from the municipal authority during emergency situations fulfills my needs.	1	5	3.29	1.147
25.	Many of my neighbors are my friends.	1	5	3.53	1.183
26.	I intend to leave my place of residence in an emergency.	1	5	2.03	1.234
27.	In an emergency, the public transportation where I live will function.	1	5	2.53	1.106
28.	Officials in my place of residence demonstrate leadership abilities.	1	5	3.28	1.118

**Table A2:** Distribution of scores for individual CCRAM factors.

CCRAM Factors	Min	Max	Mean	SD
CCRAM total score	1	5	3.46	0.73
Leadership	1	5	3.39	0.89
Collective efficacy	1	5	3.65	0.86
Preparedness	1	5	3.04	0.94
Place attachment	1	5	3.76	0.90
Social trust	1	5	3.47	0.89

**Table A3:** Correlation between CCRAM items and confidence in health services availability.

No.	CCRAM’s Items	Correlation
1	The municipal authority functions well.	0.482 **
2	There is mutual assistance and people care for one another.	0.322 **
3	My community is prepared for an emergency situation.	0.490 **
4	I am proud to tell others where I live.	0.284 **
5	Good relationships exist between the various groups.	0.286 **
6	I trust the local decision makers.	0.517 **
7	I can count on people in my community to help me in a crisis situation.	0.300 **
8	Residents are aware of their roles in an emergency situation.	0.406 **
9	I have a sense of belonging to my community.	0.297 **
10	Residents in my community trust each other.	0.287 **
11	In my community, appropriate attention is given to the needs of children.	0.419 **
12	In my community, there are people who can help me cope with an emergency situation.	0.427 **
13	There are sufficient facilities for public protection (e.g., shelters, etc.) in my community	0.324 **
14	I remain in my community for ideological reasons.	0.187 **
15	I have faith in my mayor’s ability to lead the transfer from routine to emergency management.	0.527 **
16	I have faith in my community’s ability to overcome an emergency situation.	0.424 **
17	My family and I are acquainted with the emergency system in my town (to be activated in times of emergency).	0.381 **
18	I would be sorry to leave the town where I reside.	0.235 **
19	The municipal authorities provide services fairly.	0.507 **
20	The residents are greatly involved in the community’s activities.	0.365 **
21	The residents of my community will continue to receive Municipal services even in an emergency situation.	0.600 **

Note: ** *p* = 0.001.
